# From QI-disability to QID-12: creating a brief proxy-report measure of quality of life for children with intellectual disability

**DOI:** 10.1007/s11136-026-04223-x

**Published:** 2026-04-01

**Authors:** Melissa K. Licari, Andrew J.O. Whitehouse, Natasha N. Ludwig, Mary Wojnaroski, Rebecca Hommer, Gabrielle Conecker, JayEtta Hecker, Kelly Muzyczka, Helen Leonard, Katrina J. Williams, Dinah S. Reddihough, Jenny Downs, Peter Jacoby

**Affiliations:** 1 The Kids Research Institute Australia, Centre for Child Health Research, The University of Western Australia, PO Box 855, West Perth, Perth, WA 6872 Australia; 2 Kennedy Krieger Institute, Center for Neuropsychological and Psychological Assessment/Psychiatry and Behavioral Sciences, Johns Hopkins School of Medicine, Baltimore, MD USA; 3 Department of Psychology/Psychiatry and Behavioral Health, Nationwide Children’s Hospital, Ohio State University, Columbus, OH USA; 4 Maryland & DC DeafBlind Project, Connections Beyond Sight and Sound, College Park, MD USA; 5 The Inchstone Project, Decoding Developmental Epilepsies, Washington, DC USA; 6 Clinical Sciences, Monash University, Melbourne, Australia; 7 Department of Paediatrics, Monash Children’s Hospital, Melbourne, Australia; 8 Neurodisability and Rehabilitation, Murdoch Children’s Research Institute, Melbourne, Australia; 9 Department of Paediatrics, The University of Melbourne, Melbourne, Australia; 10 Curtin School of Allied Health, Curtin University, Perth, Australia

**Keywords:** Quality of life, Intellectual disability, Children, Measurement, Short-form

## Abstract

**Purpose:**

Quality of Life Disability (QI-Disability) is a 32-item parent-report measure assessing quality of life (QOL) in children with intellectual disability across domains of physical health, positive emotions, negative emotions, social interactions, leisure and outdoors, and independence. This study aimed to develop and validate a short form for use in clinical and research settings.

**Methods:**

Caregivers of 1,699 children with intellectual disability aged 3–18 years and representing mild to profound functional impairments, completed the QI-Disability measure as part of different studies. A Genetic Algorithm (GA) was applied to select a reduced item set. The short form was evaluated against the original scale using correlational, reliability, and Rasch analyses.

**Results:**

The GA-derived 12-item set (QID-12) represented each of the six QOL domains. Correlation between QID-12 and QI-Disability total scores was high (*r* = 0.97). Internal consistency of QID-12 was acceptable (α = 0.85). Rasch analysis demonstrated good fit of all items to the partial credit model, person separation reliability was 0.84, and there was no evidence of multidimensionality (*p* > 0.99). Item targeting was appropriate across the ability spectrum. Disordered category thresholds were observed for three items, but overall psychometric performance remained satisfactory.

**Conclusion:**

QID-12 provides a valid and reliable short form of the QI-Disability. It retains coverage of the key domains of child QOL while substantially reducing respondent burden, supporting its use in both clinical practice and population research.

**Supplementary Information:**

The online version contains supplementary material available at 10.1007/s11136-026-04223-x.

## Abstract (plain language summary)

Measuring quality of life (QOL) in children with intellectual disability is typically based on parent or proxy-report. Existing QOL questionnaires are lengthy and may form part of a battery of measures that can be time consuming to complete. Thus, there is a need for briefer, psychometrically sound questionnaires that can reduce respondent burden and provide an overall measure of child QOL for some clinical or research contexts. The Quality of Life Inventory – Disability (QI-Disability) is a validated 32-item parent-report measure of children’s QOL across six domains - physical health, positive and negative emotions, social interaction, leisure and the outdoors, and independence. This study sought to develop and validate a short-form version of QI-Disability in a large sample of children with mild to profound intellectual disability. The resulting 12-item short-form (QID-12) demonstrated strong validity and reliability, capturing the six key domains of child QOL while substantially reducing respondent burden. These findings support the use of QID-12 in both clinical practice and population research taking into account that the short form is not intended to replace the full QI-Disability in all contexts.

**Supplementary Information:**

The online version contains supplementary material available at 10.1007/s11136-026-04223-x.

## Introduction

Quality of life (QOL) is a multidimensional construct that reflects an individual’s overall wellbeing within the context of culture, personal values and goals [1]. In children, QOL typically encompasses physical health, emotional wellbeing, and social connectedness, as well as aspects of personal development and activity. Valid QOL measures enable understanding of lived experience and guidance for clinical care, and inform clinical monitoring, benchmarking, and evaluation of interventions, supports, and services. For example, measures of QOL are increasingly important in therapeutic trials for neurodevelopmental and rare genetic disorders, where QOL outcomes complement biomedical measures [2, 3]. Given the unique challenges for self-report faced by many children with intellectual disability, especially for those with more severe to profound intellectual disability, caregiver-proxy reports can provide important observations to capture QOL across multiple life domains [4].

Developed from extensive qualitative data collected from parents of children with an intellectual disability, the Quality of Life Inventory – Disability (QI-Disability) [5] is a validated 32-item measure of parent-reported child QOL. The scale captures six domains: physical health, positive emotions, negative emotions, social interaction, leisure and outdoors, and independence. There is evidence for its reliability and validity across a number of etiologies of intellectual disability and associated conditions including CDKL5 deficiency disorder, Down syndrome, Rett syndrome, cerebral palsy and autism spectrum disorder [6–8]. While used extensively in research studies, e.g., [9, 10] the 32-item length may be considered time consuming for some respondents, particularly in settings where time is limited or multiple questionnaires are administered. Developing an abbreviated version of the questionnaire without sacrificing psychometric robustness has the potential to reduce respondent burden for some clinical or population research contexts.

Traditional scale reduction methods are grounded in Classical Test Theory (CTT) or Item Response Theory (IRT) [11]. CTT-based methods typically retain items with the highest factor loadings or those that most influence internal consistency (Cronbach’s alpha), while IRT approaches select items with high information value or broad coverage of difficulty levels [11]. Both approaches may prioritise internal consistency at the expense of conceptual breadth.

In recent years, optimisation algorithms inspired by natural processes have been applied to short form development. These include Ant Colony Optimisation (ACO) [12] and Genetic Algorithms (GA) [13]. The GA technique simulates natural evolution by randomly generating different short versions of a scale, including different sets of items. These sets of items are evaluated according to a specified criterion. The sets of items that are most “fit” are retained, then randomly altered into slightly different sets (akin to natural “mutations” of an organism), and then again evaluated. The process continues until the algorithm converges to a solution that, according to the specified fitness criterion, appears to be optimal and further alterations of the item sets lead to no improvement. In this context, the fitness function aims to maximise the variance in the long form scores explained by a linear combination of items while penalising the number of items used. Although this method may still favour items with high item-total correlation, it typically avoids selecting pairs of highly correlated – and therefore homogeneous or even redundant – items [14].

While CTT methods lead to short measures with high internal consistency, the GA method is more capable of preserving the conceptual breadth of the construct being measured with retained items more heterogeneous in their content [14]. Both ACO and GA have been shown to outperform CTT methods in generating efficient and psychometrically sound short versions when considering unidimensionality, reliability, sensitivity and validity [15]. The GA algorithm has been successfully applied in the abbreviation of scales including the Psychopathic Personality Inventory – Revised [16] and the Challenge of Living with Cystic Fibrosis scale [17].

The aim of this study was to develop and validate a short form of QI-Disability using a GA approach in a large sample of children with intellectual disability. We hypothesised that the short form would demonstrate comparable psychometric properties to the full version of QI-Disability while offering a brief, efficient tool for use in some clinical practice and research contexts.

## Methods

### Sources of data

Completed QI-Disability datasets were drawn from datasets collected between 2016 and 2023. These datasets included children aged three to 18 years with mild to profound intellectual disability and associated conditions. To avoid duplicates across datasets (i.e., cases present across more than one dataset), all cases were checked and any duplicates excluded. The sample sources included:

1. The Kids Research Institute Australia datasets:
  - *QI-Disability dataset evaluating the determinants of QOL*: The initial validation of QI-Disability involved primary caregivers of 5-to-18-year-old children with intellectual disability and evidence for satisfactory goodness of fit, known-group validity and demonstrated test-retest reliability [5, 18, 19]. Following initial validation, a follow-up study of 435 5-to-18-year-old children with intellectual disability across four diagnostic groups (Rett syndrome with a pathogenic variant on the *MECP2* gene [20], Down syndrome, cerebral palsy and intellectual disability, or autism spectrum disorder and intellectual disability) identified dependency in managing personal needs, eye contact and participation in the community were predictors of QOL [21]. In this dataset, approximately half of children with Down syndrome (52%) or autism and intellectual disability (53%) spoke well and were easily understood and very few were diagnosed with epilepsy whereas the majority with Rett syndrome (98%) or cerebral palsy and intellectual disability (6%) had difficulty with speech or did not use speech for communication and had received a diagnosis of epilepsy [5]. Complete QOL datasets (*n* = 418) from this study were included in the current study.
  - *CDKL5 dataset*: A questionnaire including questions on comorbidities, functional abilities and QI-Disability was completed in 2018 by parents of 129 children aged 3 to 29 years who were registered with the International CDKL5 Disorder Database [22] and had a pathogenic variant on *CDKL5* [2]. Nearly 20% used sign or spoken language, nearly a quarter were fully enterally fed, nearly two thirds had daily seizures and 70% were taking two or more antiseizure medications [22]. Predictors of poorer QOL included lack of ability to sit, use hands and communicate, and taking three or more anti-epileptic medications [22]. Seventy-six children aged 3 to 18 years from this dataset were included in the current analysis.
  - *EQ-5D-Y-5 L evaluation dataset*: A cross-sectional survey was administered to a convenience sample of 234 caregivers of 4-to-18-year-old children with intellectual disability between June 2022 and March 2023, where 36% had autism and intellectual disability, 27% had cerebral palsy and intellectual disability and 12% had Down syndrome [23]. The data were used to examine psychometric properties of a proxy report version of the EQ-5D-Y-5 L, including some comparisons with QI-Disability domain scores [23]. One hundred and nine unique individuals were included in the current analysis.
2. *Simons Searchlight dataset* (https://www.simonssearchlight.org/): Simons Searchlight is an online registry for individuals with a genetic diagnosis associated with autism and other neurodevelopmental disorders [24]. At data download (V11, released 2023), the current Simons Searchlight Gene List (https://www.simonssearchlight.org/research/what-we-study/) contained cohorts for multiple gene and copy number variants. The data collection includes developmental and behavioural surveys submitted by parents through an online study portal. QI-Disability was administered between December 2022 and March 2023. Data from 953 individuals aged 3-to-18-year-old whose genetic variant was associated with intellectual disability were included in this analysis. In this group, nearly half (45%) had received a diagnosis of epilepsy and there were 114 gene and copy number variants (e.g., 16p11.2 deletion (*n* = 105); SCN2A (*n* = 58); 16p11.2 duplication (*n* = 55); PPP2R5D (*n* = 49); Table 1).
3. *Inchstone DEE Parents Speak survey dataset*: Parent advocacy groups from developmental and epileptic encephalopathy (DEE) communities within the DEE-P Connections network (https://deepconnections.net/) invited their members to participate in an anonymous online survey between June and November 2023. Parents were eligible to complete the survey if their child had a neurodevelopmental condition and severely impaired communication. QI-Disability was completed by 242 caregivers of individuals 2 to 18 years of whom the majority did not use words for communication (72%), 62% were diagnosed with epilepsy and 27% were taking two or more antiseizure medications [25]. To avoid potential duplicates, participants with gene variants (ASXL3, CSNK2A1, GRIN1, GRIN2A, GRIN2B, SCN2A, STXBP1) represented also in the Simons Searchlight dataset were removed from the Inchstone dataset. This analysis was restricted to individuals who were three years or older providing 143 participants for the current dataset. In this group, 128 (89.5%) reported a genetic cause for their child’s condition (e.g., SCN8A (*n* = 24; FOXG1 (*n* = 18); Table 1).

### Variables

*Quality of life* - QI-Disability is a parent-report measure used to assess quality of life for children and adolescents with intellectual disability. It is a 32-item measure with responses rated on a 5-point scale. Items cluster into six domains of physical health, positive and negative emotions, social interaction, leisure and the outdoors, and independence. Scores are scaled to a 0–100-point scale, with higher scores indicating better QOL [5].

*Descriptive variables* –Descriptive variables that were common across the datasets included age, sex, diagnosis, and ability to walk (classified as independent walking or not). Genetic variant information was available for children with Rett syndrome or Down syndrome in the QI-Disability determinants, CDKL5 deficiency disorder, Simons Searchlight and Inchstone datasets.

### Statistical methods

*Short Form Development* - The Genetic algorithm (GA) was implemented using R package *GAabbreviate.*. The GA simulates natural evolution by randomly generating short versions of the scale and evaluating them according to a fitness function until an optimal solution is reached. The algorithm is designed to minimise the following fitness function [26], where

$$Cost=Ik+\sum_{i=1}^{s}{w}_{i}(1-{R}_{i}^{2})$$

‘I’ is a user-specified fixed item cost, ‘k’ is the number of items to be retained, ‘s’ is the number of subscales, ‘w_i_’ are the weights associated with each subscale and ‘R_i_^2^’ is the amount of variance in the subscale explained by a linear combination of retained item scores. We fixed the number of retained items to be 12 and, given that our retained items would form a single subscale, minimising the above fitness function reduces to a simple maximisation of the variance in total QI-Disability score explained by a subset of 12 items.

The algorithm was run 500 times with different seed values, and the final item set selected consisted of the 12 items which appeared most often in the 500 optimum solutions. We also specified a constraint that the final item set had to contain at least one item from each QI-Disability domain and no more than 3 items from any domain.

*Short Form Psychometric Evaluation* - Pearson correlation coefficient was used to compare the 12-item total score with the QI-Disability 32-item total score. Internal consistency of the reduced item set was assessed with Cronbach’s Alpha.

Rasch analysis of the final item set was performed using the partial credit model within the R package *eRm.* Item fit to the Rasch model was assessed using Infit and Outfit mean square statistics; person separation reliability was calculated and the Martin-Loef test was used to confirm unidimensionality of the reduced item set. Targeting of the items was assessed using the Person-Item map showing the correspondence between item difficulties and person abilities. Finally Disordered Category Thresholds for any item were noted. Disordered thresholds occur when the ordering of the thresholds does not correspond to the natural order of difficulty of the categories.

## Results

A total of 1,699 unique and complete QI-Disability measures were included. The median age was 9.4 years (range 3.0–18.3 years). The country of residence was known for participants in all datasets except for the Simons Searchlight dataset (*n* = 953; a global database where anonymous data are provided). Among the remaining participants, 539 were from Oceania, with the majority from Australia; 160 were from North America, with most from the United States; 38 were from Europe, with the largest group from Germany (*n* = 10); and small numbers were from South America, Africa, and Asia.

The mean (SD) QI-Disability total score was 69.2 (14.4) (Table 1). Participant characteristics varied across cohorts, including differences in diagnosis, sex distribution, ability to walk, and QI-Disability scores. Across datasets, the sample included children with a wide range of functional abilities, including variation in mobility, communication, and independence, consistent with mild to profound intellectual disability and a range of adaptive behaviour abilities. For example, most children with autism were male (75.7%), most with CDD were female (82.9%) and all children with RTT were female (100%). Most children in the Simons dataset (88.1%), with autism and intellectual disability (98.0%) and all with Down syndrome could walk independently. Smaller fractions of children with cerebral palsy and intellectual disability (32.0%), CDD (32.0%), RTT (26.5%) and in the Inchstone dataset (37.3%) could walk independently. Higher QI-Disability Total and QID-12 scores were observed for children with Down syndrome and lower QI-Disability Total and QID-12 scores were observed for children with a genetically caused epilepsy condition including CDD and many of those in the Inchstone dataset. See Table 1.

**Table 1:** Sample characteristics by data source (*n* = 1,699)

	Simons Searchlight^ (*N* = 953)	The Kids (*N* = 603)						Inchstone DEE Parents Speak^#^ (*N* = 143)
		Autism (*N* = 152)	CDD (*N* = 76)	CP (*N* = 180)	Down syndrome (*N* = 94)	Rett syndrome (*N* = 68)	Other (*N* = 33)	
Age Median (Range)	8.5 (3.0–17.9)	10.9 (4.7–17.6)	10.2 (5.0–18.0)	12.3 (5.9–18.0)	9.5 (5.1–17.8)	11.2 (5.0–17.9)	10.1 (5.0–18.2)	8.4 (3.0–18.3)
Sex Male (%)	535 (56.1)	115 (75.7)	13 (17.1)	106 (58.9)	39 (41.5)		17 (51.5)	65 (45.5)
Female (%)	418 (43.9)	37 (24.3)	63 (82.9)	74 (41.1)	55 (58.5)	68 (100)	16 (48.5)	78 (54.6)
Walks independently N (%)	804 (88.1)	149 (98.0)	24 (32.0)	66 (36.7)	94 (100.0)	18 (26.5)	24 (72.7)	53 (37.3)
QI-Disability Total scores Mean (SD)	71.5 (14.4)	67.7 (11.1)	60.2 (15.0)	66.5 (13.3)	77.8 (10.4)	66.0 (11.3)	67.8 (17.7)	60.2 (14.4)
QID-12 scores Mean (SD)	69.9 (15.7)	67.9 (12.2)	58.6 (17.3)	64.0 (14.9)	77.4 (11.7)	63.1 (12.6)	66.3 (18.0)	58.2 (16.2)

^ Gene diagnoses in the Simons Searchlight dataset: 16p11.2 deletion (*n* = 105); SCN2A (*n* = 58); 16p11.2 duplication (*n* = 55); PPP2R5D (*n* = 49); STXBP1 (*n* = 39); SLC6A1 (*n* = 37); CSNK2A1 (*n* = 35); CTNNB1 (*n* = 35); MED13L (*n* = 35); GRIN2B (*n* = 33); 1q21.1 duplication (*n* = 26); ASXL3 (*n* = 25); 1q21.1 deletion (*n* = 22); SYNGAP1 (*n* = 21); DLG4 (*n* = 17); HIVEP2 (*n* = 17); SETBP1 (*n* = 17); DISTAL 16p11.2 duplication (*n* = 14); HNRNPH2 (*n* = 12); CHAMP1 (*n* = 11); DYRK1A (*n* = 11); DISTAL 16p11.2 deletion (*n* = 10); 15q11.2 BP1-BP2 deletion (*n* = 9); 7q11.23 duplication (*n* = 9); AUTS2 (*n* = 8); CHD2 (*n* = 8); PACS1 (*n* = 8); VPS13B (*n* = 8); MED13 (*n* = 7); WDFY3 (*n* = 7); ANKRD11 (*n* = 6); ARID1B (*n* = 6); CSNK2B (*n* = 6); SETD5 (*n* = 6); ADNP (*n* = 5); FOXP1 (*n* = 5); KMT2E (*n* = 5); TRIO (*n* = 5); TRIP12 (*n* = 5); and 75 variants with frequency < 5.

^#^ Parent reported gene diagnoses in the Inchstone dataset: Phelan McDermid syndrome (*n* = 31); SCN8A (*n* = 24); FOXG1 (*N* = 18); ASXL1 (*N* = 12); DUP15Q (*N* = 6); KCNT1 (*N* = 6); CACNA1A (*N* = 5); and 26 gene variants with frequency < 5. No genetic diagnosis reported for 15 individuals.

After applying the genetic scale reduction algorithm and domain membership restrictions, the most frequently selected items comprising the final QID-12 include two items from the Physical Health domain, three items from the Positive Emotions domain, one item each from the Negative Emotions and Social Interactions domain, three items from the Leisure and the Outdoors domain and two items from the Independence domain (Table 2).

**Table 2:** Fit Statistics and item locations for the items in the reduced item set

Domain	Item	Outfit Mean Square*	Infit Mean Square*	Item Location (logits)
Physical health	Had enough energy to participate in routines and activities	0.97	0.921	-0.167
	Slept well through the night	1.213	1.16	0.664
Positive emotions	Been in a good mood	0.765	0.777	-0.561
	Smiled or brightened their facial expression	0.697	0.738	-0.369
	Showed happiness through body language	0.637	0.671	-0.143
Negative emotions	Appeared upset or angry	1.433	1.407	1.215
Social interactions	Appeared relaxed when making eye contact	0.888	0.887	0.712
Leisure and the outdoors	Enjoyed feeling steady or stable during physical activities	0.809	0.821	0.802
	Enjoyed physical activities	0.784	0.822	0.449
	Enjoyed going on outings in the community	0.914	0.912	0.758
Independence	Made their own choices for activities or things they enjoy	1.053	1.039	0.822
	Helped to complete routine activities	1.086	1.092	1.450

*Satisfactory model fit indicated by mean square values below 1.5

Correlation of the 12-item total with the QI-Disability total score (average of domain scores) was high (Pearson’s rho = 0.970), demonstrating that the short form reflected overall QOL. Internal consistency (Alpha = 0.84) was satisfactory, supporting the reliability of the reduced dataset.

**Fig. 1:**
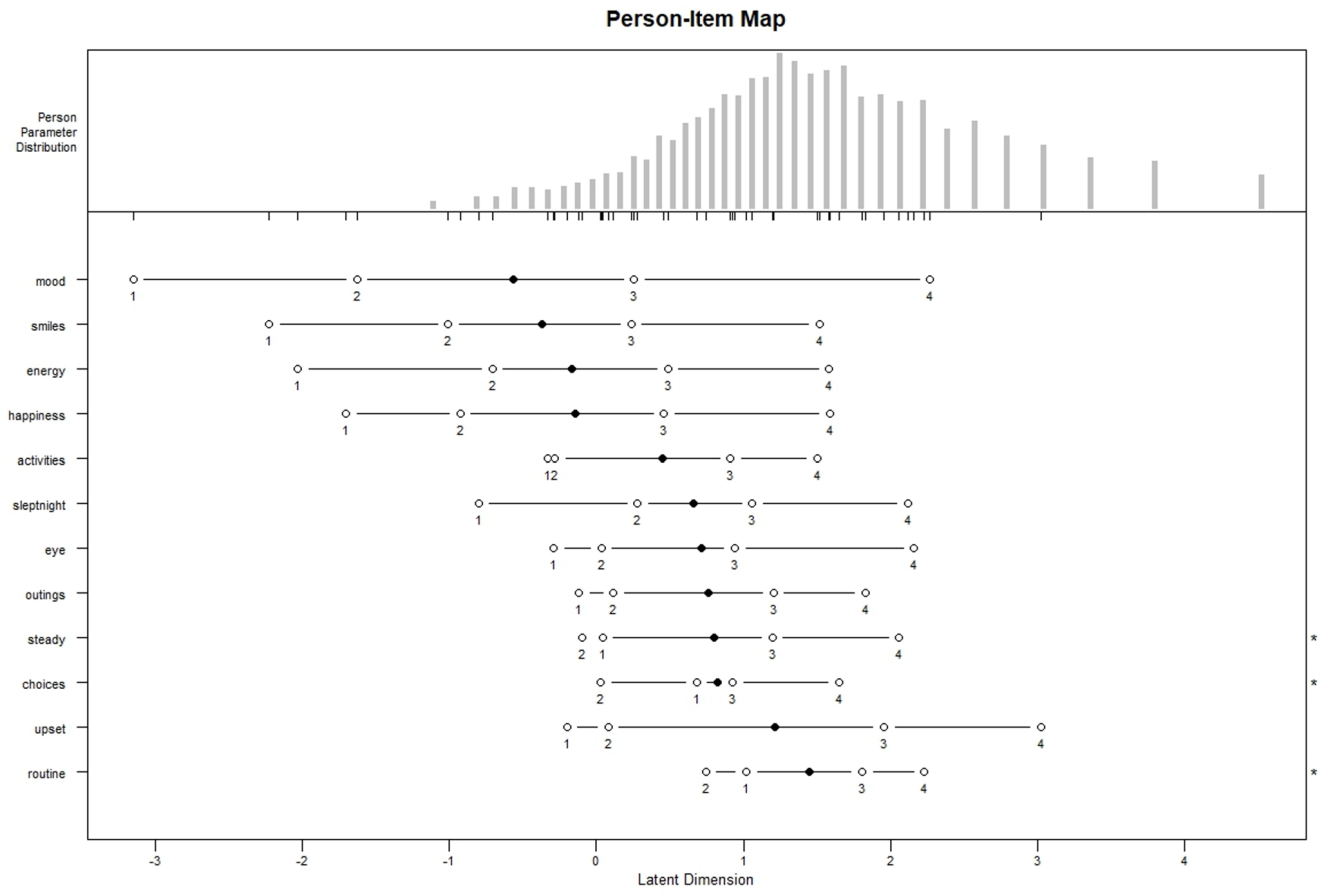
**Person-Item map for the reduced QI-Disability item set**

Rasch analysis of the QID-12 confirmed satisfactory fit to the partial credit model, with both Infit and Outfit mean square statistics all below the recommended upper threshold of 1.5. (Table 2). Person separation reliability was 0.84, and the Martin-Loef test showed no significant departure from unidimensionalty (*p* = 0.12). The Person Item map (Fig. 1) showed reasonable targeting with the item difficulty locations covering the full range of ability scores in the sample although there was better coverage at the lower end of the quality of life score spectrum. Minor irregularities in category thresholds were observed for the items ‘feeling steady or stable during physical activities’, ’making their own choices’ and ‘helped with routines’ where the difficulty thresholds between categories 1 and 2 (‘Never’ and ‘Rarely’) and categories 2 and 3 (‘Rarely and ‘Sometimes’) were reversed from their natural order.

## Discussion

This study developed and validated a 12-item short form of QI-Disability, referred to as QID-12. The Genetic Algorithm (GA) has been shown to outperform other techniques for scale reduction and has been successfully applied by researchers to abbreviate scales in a variety of medical and psychological settings [17, 18]. Using a GA approach in a large and diverse sample of 1,699 children with intellectual disability, we systematically identified a reduced item set that retained strong alignment with the original 32-item scale. The QID-12 showed high correlation with the original measure, acceptable internal consistency, and good Rasch model fit, confirming that it provides an accurate representation of overall quality of life.

The final 12 items spanned the six domains of the original measure to preserve the conceptual breadth of QOL as operationalised by QI-Disability. Correlation with the full scale was high (0.97) demonstrating that QID-12 scores reflected overall QOL scores, observed also in the score patterns for different groups with the dataset. While the high correlation between QID-12 and the full QI-Disability is reassuring, it should be interpreted in context. The short form draws directly from the original 32-item measure and was developed within the same sample, which likely inflates the association. Reliability estimates, although slightly reduced compared to the full scale, remained within acceptable ranges (α = 0.85; person separation reliability = 0.84). Rasch analysis further confirmed the psychometric adequacy of the short form, with satisfactory item fit, and item difficulty reasonably well distributed to capture the full range of children’s quality of life, along with evidence of unidimensionality. Although quality of life is multidimensional, the absence of strong evidence against unidimensionality supports the use of the QID-12 as an index of overall quality of life rather than a domain-level assessment tool. Although three items (‘steady stable’, ‘choices’ and ‘helped routine’) showed slight inconsistencies in response category ordering, it has been shown that this phenomenon occurs when there is a low frequency of intermediate response categories and that it probably does not compromise the utility of a measurement scale [27].

The QID-12 has clear utility in settings where respondent burden is a concern, such as large-scale surveys, longitudinal studies, registries, and clinical settings where caregivers are asked to complete multiple measures. In these contexts, the availability of a brief and psychometrically robust index of overall quality of life will support the inclusion of patient and caregiver-reported outcomes alongside other measures, improving feasibility without compromising measurement integrity. In clinical settings, the QID-12 may be useful as a quick screening and monitoring tool to track overall quality of life over time or the effectiveness of interventions and services. However, it is not intended to replace the full QI-Disability in situations where detailed domain-level assessment is required. While the short form offers a more efficient way to assess QOL, the longer version of the QI-Disability still holds distinct advantages. The full 32-item measure covers a wide range of questions, providing a more comprehensive understanding of a child’s QOL in certain contexts. Although the short forms reduced length is beneficial in some situations, it may miss acumen for each of the dimensions when more detailed information is required. Specifically, in clinical settings where more detailed insight into a child’s specific QOL-challenges is needed, the full version provides more granular insights. Therefore, it is important not to overlook the original 32-item measure in these instances.

Strengths of this study include the large, heterogeneous sample, which included data from multiple sources and spanned a broad age range and functional abilities. The use of an advanced optimisation method also represents a methodological strength, allowing for systematic identification of an item set that maintains conceptual breadth whilst reducing length. One limitation is that a few items exhibited disordered category thresholds although it unlikely impacted the overall psychometric performance of a scale when disordered thresholds are an artefact of a low frequency of intermediate response categories [27]. There were also limited descriptive variables that were common across the data sources, although more sample description is provided in each of the accompanying papers. Additionally, while the QID-12 was validated against the full version, future research should focus on validating the QID-12 in independent cohorts and across more diverse settings. This would help confirm the tool’s generalizability and assess whether it performs the same way as the full version in different populations and contexts.

## Conclusions

The QID-12 presents a psychometrically sound and efficient short form of QI-Disability. It provides clinicians and researchers with a practical tool for measuring child QOL that minimises burden while maintaining conceptual breadth and measurement integrity. Future research should focus on further validation of the QID-12 in independent cohorts, examining its responsiveness to change in clinical trials, and comparing its performance to the full 32-item version across diverse settings.

## Supplementary Information

Below is the link to the electronic supplementary material.

Supplementary Material 1

## Data Availability

The data supporting the findings of this study were obtained from multiple studies and are subject to ethical and governance restrictions. As these data include sensitive information relating to children with intellectual disability, they are not publicly available. De-identified data may be made available upon reasonable request to the corresponding author, subject to approval by the relevant ethics committees and data custodians.
