# Supplementary material for: From QI-disability to QID-12: creating a brief proxy-report measure of quality of life for children with intellectual disability
Source: Qual Life Res. 2026 Apr 1;35(5):112. doi: 10.1007/s11136-026-04223-x (PMC13043590; doi:10.1007/s11136-026-04223-x)
Supplement: Supplementary file 1 — Supplementary Material 1 [file 11136_2026_4223_MOESM1_ESM.docx]

Title

From QI-Disability to QID-12: Creating a Brief Proxy-Report Measure of Quality of Life for Children with Intellectual Disability

Journal

Quality of Life Research

Author Information

Melissa K. Licari^1^

Andrew J.O. Whitehouse^1^

Natasha N. Ludwig^2^

Mary Wojnaroski^3^

Rebecca Hommer^3^

Gabrielle Conecker^4^

JayEtta Hecker^4^

Kelly Muzyczka^4^

Helen Leonard^1^

Katrina Williams^5,6^

Dinah S. Reddihough^7,8^

Jenny Downs^1,9*^

Peter Jacoby^1*^

*Senior Authors

Affiliations

1. The Kids Research Institute Australia, Centre for Child Health Research, The University of Western Australia, Perth, Australia.

2. Kennedy Krieger Institute/Johns Hopkins School of Medicine, Center for Neuropsychological and Psychological Assessment/Psychiatry and Behavioral Sciences, Baltimore, MD, USA.

3. Nationwide Children’s Hospital/Ohio State University, Department of Psychology/Psychiatry and Behavioral Health, Columbus, OH, USA.

4. The Inchstone Project, Decoding Developmental Epilepsies, Washington, DC.

5. Clinical Sciences, Monash University, Melbourne, Australia

6. Department of Paediatrics, Monash Children's Hospital, Melbourne, Australia

7. Neurodisability and Rehabilitation, Murdoch Children's Research Institute, Melbourne, Australia.

8. Department of Paediatrics, The University of Melbourne, Melbourne, Australia

9. Curtin School of Allied Health, Curtin University, Perth, Australia.

Corresponding author

Professor Jenny Downs

The Kids Research Institute Australia

University of Western Australia

PO Box 855, West Perth, Western Australia, 6872, Australia

Tel: +61 8 6319 1000

Email: Jenny.Downs@thekids.org.au

ORCID numbers

Melissa Licari - 0000-0003-3705-5323

Andrew Whitehouse - 0000-0001-8722-1575

Natasha N. Ludwig - 0000-0002-8934-7267

Mary Wojnaroski - 0009-0006-5595-0419

Rebecca Hommer - 0009-0005-4800-6182

Gabrielle Conecker - 0000-0003-3274-0292

JayEtta Hecker - 0009-0001-2704-5044

Kelly Muzyczka - 0000-0001-5307-0692

Helen Leonard - 0000-0001-6405-583

Katrina J. Williams - 0000-0002-1686-4458

Dinah Reddihough - 0000-0003-3634-7906

Jenny Downs - 0000-0001-7358-9037

Peter Jacoby - 0000-0001-5092-7697

**Supplementary Table –** Order of inclusion of all QI-Disability items in selected 12-item subset

| **Item summaries** | **Domain** | **No. Hits*** |
| --- | --- | --- |
| **Slept well through the night** | **Physical Health** | 491 |
| **Had enough energy to participate in routines and activities** | **Physical Health** | 420 |
| **Made their own choices for activities or things they enjoy** | **Independence** | 411 |
| **Enjoyed going on outings in the community** | **Leisure & the Outdoors** | 369 |
| **Helped to complete routine activities** | **Independence** | 348 |
| **Been in a good mood** | **Positive Emotions** | 337 |
| **Showed happiness through body language** | **Positive Emotions** | 326 |
| **Appeared relaxed when making eye contact** | **Social Interactions** | 318 |
| **Smiled or brightened their facial expression** | **Positive Emotions** | 311 |
| **Enjoyed physical activities** | **Leisure & the Outdoors** | 270 |
| Showed cheeky or comical mannerisms | Positive Emotions | 265 |
| **Enjoyed feeling steady or stable during physical activities** | **Leisure & the Outdoors** | 254 |
| Enjoyed being included | Social Interactions | 233 |
| **Appeared upset or angry** | **Negative Emotions** | 224 |
| Been unsettled without apparent reason | Negative Emotions | 198 |
| Responded positively when others paid attention to them | Social Interactions | 172 |
| Enjoyed making things with their hands—can be with help | Independence | 154 |
| Enjoyed spending time outdoors | Leisure & the Outdoors | 124 |
| Showed pleasure or excitement when looking forward to activities | Social Interactions | 120 |
| Showed signs of being anxious or agitated | Negative Emotions | 103 |
| Enjoyed moving their body | Leisure & the Outdoors | 102 |
| Expressed happiness when understood | Social Interactions | 70 |
| Initiated greetings with people verbally | Social Interactions | 69 |
| Expressed their needs | Independence | 64 |
| Been alert and aware during the day | Physical Health | 62 |
| Expressed discomfort with changes in routine | Negative Emotions | 48 |
| Became withdrawn with a low mood | Negative Emotions | 47 |
| Enjoyed social experiences of mealtimes | Social Interactions | 34 |
| Kept in good general health | Physical Health | 26 |
| Enjoyed using technology | Independence | 19 |
| Showed aggression | Negative Emotions | 10 |
| Deliberately hurt themselves | Negative Emotions | 1 |

* Number of runs (ex 500) in which the item was included among the 12 selected by the genetic algorithm. Items in bold included in QID-12 after applying the domain membership restriction where we sought to include at least one item from each domain.
